# Procalcitonin as a Predictive Marker of Incident Liver Disease

**DOI:** 10.1111/liv.70132

**Published:** 2025-05-12

**Authors:** Amanda Finnberg‐Kim, Mats Pihlsgård, Kristina Önnerhag, Olle Melander, Sofia Enhörning

**Affiliations:** ^1^ Department of Clinical Sciences in Malmö Lund University Malmö Sweden; ^2^ Department of Gastroenterology and Hepatology Skåne University Hospital Malmö Sweden; ^3^ Perinatal and Cardiovascular Epidemiology, Lund University Diabetes Centre Department of Clinical Sciences in Malmö, Lund University Malmö Sweden; ^4^ Department of Internal Medicine Skåne University Hospital Malmö Sweden

**Keywords:** C‐reactive protein, liver cirrhosis, liver disease, procalcitonin, risk assessment

## Abstract

**Background and Aims:**

Previous studies have shown that procalcitonin (PCT) concentration is elevated in patients with liver disease without evidence of bacterial infection. We aimed to investigate the association between elevated PCT and the future risk of liver disease.

**Method:**

PCT was measured in 3897 individuals without known liver disease in the Malmö Diet and Cancer Cardiovascular Cohort (MDC‐CC) and in 3854 individuals in the Malmö Preventive Project cohort (MPP). Cox proportional hazards regression models were used to analyse the risk of register‐verified incident liver disease by PCT levels. We performed our analyses in a pooled sample of both the MPP and MDC‐CC cohorts, as well as separate analyses for each cohort.

**Results:**

70 subjects in MDC‐CC and 49 subjects in MPP were diagnosed with non‐viral liver disease during a median follow‐up of 27.1 and 14.8 years, respectively. In multivariate adjusted models in the pooled sample, individuals with high PCT (> 0.05 ng/mL) had a significantly increased risk of developing liver disease compared to subjects with PCT concentrations below the cutoff (hazard ratio (HR) 3.4, 95% confidence interval (CI) 2.07–5.63, *p* < 0.001). The HR per standard deviation increase of log‐transformed PCT was 1.56 (95% CI 1.32–1.85, *p* < 0.001) in multivariate adjusted models. Separate cohort‐specific sensitivity analyses, including additional adjustment for C‐reactive protein, showed similar effect estimates as the pooled analyses.

**Conclusions:**

Elevated concentration of PCT independently predicts non‐viral liver disease. These findings could have implications for risk assessment but also highlight the possibility of PCT as a direct cause of hepatocyte damage.

**Summary**

- Procalcitonin (PCT) is used as a biomarker for bacterial infections, but PCT is also elevated in other conditions such as both acute and chronic liver diseases.
- The relationship between elevated PCT and liver damage has been suggested to be linked to systemic inflammation, but it is also possible that PCT has a direct toxic effect on hepatocytes.
- In a population‐based sample, we found PCT concentrations in plasma to predict the development of advanced chronic liver disease several years later.
- In selected patients, PCT could potentially be used to detect liver disease at an earlier stage, thereby achieving timely treatment of the underlying cause of liver injury.

**Abbreviations**
BMI: body mass index
CI: confidence interval
CKD‐EPI: CKD Epidemiology Collaboration
CRP: C‐reactive protein
DAMPs: damage‐associated molecular pathways
eGFR: estimated glomerular filtration rate
HDL: high‐density lipoprotein cholesterol
HR: hazard ratio
ICD: International Classification of Diseases
LDL: low‐density lipoprotein cholesterol
MASLD: metabolic dysfunction‐associated steatotic liver disease
MDC‐CC: the Malmö Diet and Cancer Cardiovascular Cohort
MPP: the Malmö Preventive Project cohort
PAMPs: pathogen‐associated molecular patterns
PCT: procalcitonin
SD: standard deviation

## 1 Introduction

Liver disease is the cause of approximately 2 million deaths per year worldwide [1]. Vaccination programmes and increased access to anti‐viral drugs are expected to affect the prevalence and improve the outcome of viral hepatitis [2]. However, the prevalence and complications of alcohol‐associated liver disease and metabolic dysfunction‐associated steatotic liver disease (MASLD) are increasing. MASLD was formerly described as non‐alcoholic fatty liver disease, which is now the most common liver disorder in the Western world [3]. The global burden of liver disease will continue to be substantial, and since the progression to liver cirrhosis in many cases can be prevented, there is a need for better ways to predict who is at risk.

Procalcitonin (PCT) is widely used as a biomarker for bacterial infections. It is a precursor peptide of the thyroid hormone calcitonin, and it is mainly produced by thyroid C‐cells. In healthy individuals, almost no PCT is released into the circulation, and levels remain very low [4, 5]. During inflammation, particularly as a response to bacterial infection, various cells and tissues, including the liver, produce PCT, and concentrations in the bloodstream increase significantly. Non‐infectious conditions associated with elevated concentrations of PCT include extensive surgery, burns, multitrauma, pancreatitis, neuroendocrine tumours, and liver disease [6, 7]. Elevated PCT within the normal range has also been associated with components of the metabolic syndrome [8].

Previous studies have shown that PCT concentrations are elevated above the normal range in patients with acute liver failure despite the absence of bacterial infection [9, 10]. Also, in advanced chronic liver disease, PCT concentrations are elevated without evidence of infection [9, 11]. The relationship between elevated PCT and liver damage is not completely understood, but different mechanisms have been suggested, such as bacterial translocation from the gut into the portal circulation as well as hepatocyte damage leading to a local inflammatory response in the liver, both leading to systemic inflammation [12, 13]. PCT has also been suggested to have a direct toxic effect on hepatocytes [14].

We hypothesise that plasma PCT concentrations may be elevated before advanced chronic liver disease has developed in subjects not yet diagnosed with liver disease at baseline.

## 2 Methods

### 2.1 Population

In this study, individuals participating in the Swedish Malmö Diet and Cancer Cardiovascular Cohort (MCD‐CC) and the Malmö Preventive Project cohort (MPP) were included.

The Malmö Diet and Cancer study is a population‐based prospective cohort of 30 447 individuals from the city of Malmö, Sweden, who were recruited between 1991 and 1996 with the aim to investigate how dietary patterns influence cancer. The participants underwent a physical examination, laboratory tests, and a self‐administered questionnaire [15]. MDC‐CC refers to a sub‐study of 6103 randomly selected individuals from the original cohort, of which the aim was to study the epidemiology of carotid artery disease. In this subsample, 5540 individuals agreed to reschedule a visit for fasting plasma sampling, which were collected and frozen to −80°C for later analyses. PCT was successfully measured in 4192 of the MDC‐CC participants, and complete data on all covariables were available for 3897 individuals who were all selected for further analyses.

MPP is another population‐based prospective cohort and consists of 33 346 individuals who resided in the city of Malmö, Sweden, and who were recruited between 1974 and 1992. The participants were screened for risk factors for all‐cause mortality and cardiovascular disease and underwent a physical examination, laboratory tests, and a self‐administered questionnaire. Participants who were alive between 2002 and 2006 were invited to a re‐examination (*n* = 18 240). Risk factors were reassessed, and fasting plasma samples were frozen to −80°C for later analyses [16, 17]. From this sample, 5410 individuals were randomly selected for additional laboratory measurement in stored plasma. The only prerequisite that existed before an individual could be a subject for random selection was that they had not been participating in MDC‐CC. Within the randomly selected sample, PCT was successfully measured in 5289 individuals, and complete data on all covariables were available for 3854 individuals who were all selected for further analyses.

The research was conducted in accordance with the Declaration of Helsinki, and study protocols were approved by the regional Ethics Committee of Lund University, Dnr. LU 51–90 and 85/2004. All participants provided written informed consent.

### 2.2 Data Collection and Laboratory Measurements

Blood pressure was measured after 10 min of rest in a supine position. Hypertension was defined as systolic blood pressure of ≥ 140 mmHg, diastolic blood pressure of ≥ 90 mmHg, or use of antihypertensive medication.

Diabetes was defined as a fasting plasma glucose of ≥ 7.0 mmol/L (in MPP) or fasting blood glucose of ≥ 6.1 mmol/L (in MDC‐CC), self‐reported physician diagnosis of diabetes, or use of antidiabetic medication.

Alcohol consumption was assessed in both cohorts. In MDC‐CC a daily consumption in grams was calculated from participants’ food diaries and converted to grams per week. In MPP, the present alcohol intake was assessed by a questionnaire where the participants answered how many bottles/cans of beer, or bottles of wine, or centilitres of distilled spirits (40% alcohol) they consumed per week. In Sweden, a standard drink is equal to 12 g of alcohol. We assumed a bottle/can of beer was equal to 1 standard drink, a bottle of wine equal to 5 standard drinks, and 4 cL of distilled spirits equal to 1 standard drink. Based on these assumptions, the number of drinks per week was converted to grams of alcohol per week. Hazardous alcohol consumption was defined as > 108 g/week for women and > 168 g/week for men according to recent Swedish guidelines [18].

Smoking was defined as any cigarette smoking within the past year and assessed by a self‐administered questionnaire.

C‐reactive protein (CRP) was measured using a high‐sensitivity assay (Tina‐quant CRP latex assay, Roche Diagnostics, Basel, Switzerland) on an ADVIA 1650 Chemistry System (Bayer Healthcare, NY, USA). The average coefficient of variation was 4.59 [19].

PCT was measured by an ultrasensitive immunoflourescence assay (PCT sensitive LIA; BRAHMS GmbH, Hennigsdorf, Germany). The lower detection limit was 0.006 ng/mL, and the functional assay sensitivity was 0.007 ng/mL [20, 21]. High PCT was defined as > 0.05 ng/mL, which is in line with the established reference limit used in clinical practice [22].

Total cholesterol, high‐density lipoprotein cholesterol (HDL), triglycerides, and creatinine were measured according to standard procedures at the Department of Clinical Chemistry, Skåne University Hospital, Malmö. Low‐density lipoprotein cholesterol (LDL) was calculated using the Friedewald formula. The 2009 CKD Epidemiology Collaboration (CKD‐EPI) creatinine formula was used to calculate eGFR [23].

### 2.3 Endpoints

Individuals with liver disease were identified by the Swedish National Patient Register, which is a register that covers almost all hospital discharges and hospital‐based outpatient care in Sweden, and the Swedish Cause‐of‐death Register, which covers all deaths among Swedish residents, including deaths that occur abroad [24].

Prevalent and incident liver disease was defined based on any of the following International Classification of Diseases (ICD) 9 codes: 571.0–571.6, 571.8–571.9, 576.1, or ICD 10 codes: B18, K70.0–K70.4, K70.9, K72.1, K72.9, K73.0–K73.2, K73.8–K73.9, K74.0–K74.6, K75.2–K75.4, K75.8–K75.9, K76.0, K76.8, K83.0A, which included diagnoses of MASLD, fibrosis, cirrhosis, alcohol‐associated liver disease, chronic cholestatic liver disease, inflammatory liver disease, chronic viral hepatitis, and chronic liver failure. Individuals with prevalent or incident toxic liver disease, acute viral hepatitis, or acute liver failure were excluded from the analysis due to the acute onset of illness.

Due to the low number of incident liver disease cases in both cohorts, we investigated groups of diagnoses together. The diagnosis groups were formed based on similarity regarding pathophysiology and/or aetiology. Chronic viral hepatitis was seen as a separate group of diagnoses since it is a viral infection. We identified the incidence of chronic viral hepatitis in both cohorts, but we did not include the diagnosis in our final analyses since we wanted to investigate PCT as a predictor of liver disease in the absence of both bacterial and viral infections. We performed our analyses in a pooled sample of both the MPP and MDC‐CC cohorts. Individuals with a diagnosis of liver disease at baseline were excluded from analyses. We also performed separate analyses for each cohort.

### 2.4 Statistics

PCT was skewed to the right and subsequently logarithmically transformed. Both the standard deviation (SD) of log‐transformed PCT, elevated PCT (> 0.05 ng/mL) and quartiles of PCT were used in separate models and related to the risk of developing liver disease by using multivariate Cox proportional hazards models and Kaplan–Meier analyses. Differences in risk in the Kaplan–Meier analyses were evaluated using the log‐rank test. The multivariate regression analyses were adjusted for age, gender, body mass index (BMI), hazardous alcohol consumption, total cholesterol, LDL, HDL, triglycerides, lipid‐lowering treatment, prevalent diabetes, creatinine, prevalent hypertension, and smoking. In a separate subgroup analysis in MDC‐CC, we also adjusted for CRP. Interaction terms (log‐transformed PCT × gender, log‐transformed PCT × BMI and log‐transformed PCT × smoking, respectively) were used to ascertain interaction between PCT and these variables on liver disease development. The interaction tests were conducted by entering the interaction term into the multivariate regression model together with log‐transformed PCT and all other covariates listed above.

A two‐sided *p*‐value of < 0.05 was considered statistically significant. SPSS statistical software (version 29.0; SPSS Inc., Chicago, IL, USA) was used for all calculations.

## 3 Results

In MPP, 25 subjects (0.6%) had a diagnosis of prevalent liver disease at baseline, and the corresponding number in MDC‐CC was 9 (0.2%) individuals. The mean age at baseline in MPP was 69 ± 6 years; 72% were male and 21% had PCT levels above 0.05 ng/mL. In MDC‐CC, the mean age at baseline was 58 ± 6 years; 42% were male and 1% had PCT levels above 0.05 ng/mL. Baseline characteristics of the population are shown in Table 1.

**TABLE 1:** Baseline characteristics of the study population.

Subjects without prevalent liver disease of any type at baseline	MPP (*n* = 3854) *n* = 3829	MDC‐CC (*n* = 3897) *n* = 3888
Age, mean (SD) years	69.1 (6.3)	57.6 (6.0)
Gender, men, *n* (%)	2772 (72.4)	1615 (41.5)
Body mass index, mean (SD), kg/cm^2^	27.2 (4.2)	25.8 (3.9)
Smokers, *n* (%)	733 (19.1)	1032 (26.5)
Hazardous alcohol consumption *n* (%)^a^	702 (18.3)	622 (16.0)
p‐glucose, mean (SD), mmol/L	5,9 (1.5)	NA
b‐glucose, mean (SD), mmol/L	NA	5.2 (1.4)
Prevalent diabetes, *n* (%)	472 (12.3)	365 (9.4)
p‐triglycerides, mean (SD), mmol/L	1.25 (0.6)	1.31 (0.6)
High‐density lipoprotein, mean (SD), mmol/L	1.37 (0.4)	1.39 (0.4)
Low‐density lipoprotein, mean (SD), mmol/L	3.6 (1.0)	4.17 (1.0)
Lipid lowering treatment, *n* (%)	196 (5.1)	93 (2.4)
Systolic blood pressure, mean (SD), mmHg	145.9 (20.3)	141.9 (19.0)
Diastolic blood pressure, mean (SD), mmHg	84.0 (10.7)	87.0 (9.5)
Prevalent hypertension, *n* (%)	2854 (74.5)	2505 (64.4)
Antihypertensive treatment *n* (%)	1514 (39.6)	669 (17.2)
Creatinine, mean (SD), μmol/L	97.9 (28.5)	83.9 (15.8)
eGFR, mean (SD), mL/min/1,73 m^2^	66.4 (15.5)	76.9 (13.5)
p‐CRP, median (25th; 75th percentile), mg/L^b^	NA	1.3 (0.6–2.8)
p‐PCT, median (25th; 75th percentile), ng/mL	0.035 (0.025–0.047)	0.016 (0.013–0.020)
High p‐PCT *n* (%)^c^	794 (20.7)	47 (1.2)

Abbreviations: CRP, C‐reactive protein; eGFR, estimated glomerular filtration rate; MDC‐CC, Malmö Diet and Cancer Cardiovascular Cohort; MPP, Malmö Preventive Project; PCT, procalcitonin; SD, standard deviation.

^a^ Alcohol consumption exceeding 168 g per week (men) and 108 g per week (women).

^b^ CRP is only available in MDC‐CC, *n* = 3852.

^c^ High PCT defined as PCT > 0.05 ng/mL.

The incident liver diseases in subjects free from any known liver disease at baseline are shown in Table 2. In MPP, 49 individuals not diagnosed with liver disease at baseline developed liver disease of any type except for chronic viral hepatitis during a median follow‐up time of 14.8 years (11.3–15.7, 25th—75th percentile). In MDC‐CC, 70 individuals developed non‐viral liver disease during a median follow‐up time of 27.1 years (19.6–28.1, 25th—75th percentile). Both cohorts had low numbers of incident liver disease, but fibrosis/cirrhosis of the liver of unknown origin, non‐specific liver failure, alcohol‐associated liver disease, and chronic viral hepatitis were slightly more common than other diagnoses in both MPP and MDC‐CC.

**TABLE 2:** Number of individuals with incident liver disease diagnoses in subjects free from any type of liver disease at baseline.

Disease	MPP cohort *n* = 3829	MDC‐CC cohort *n* = 3888
Fibrosis/cirrhosis of the liver of unknown origin	15	25
Non‐specified liver failure	20	15
Alcohol‐associated liver disease	13	15
Chronic viral hepatitis	10	13
MASLD	5	9
Chronic cholestatic liver disease	4	13
Inflammatory/autoimmune liver disease	5	6
Chronic hepatitis not classified elsewhere	1	4
Non‐specified chronic hepatitis	1	4
Other specified diseases of the liver	3	8

*Note:* There is an overlap between different diagnoses.

Abbreviations: MASLD, Metabolic dysfunction‐associated steatotic liver disease; MDC‐CC, Malmö Diet and Cancer Cardiovascular Cohort; MPP, Malmö Preventive Project.

**FIGURE 1:**
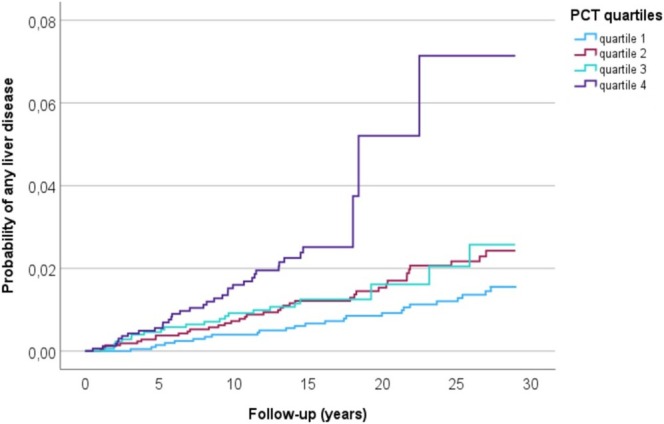
Kaplan–Meier curve showing increased probability of any type of incident non‐viral liver disease in individuals in the MDC‐CC and MPP cohorts with increasing PCT concentration (split into quartiles where quartile 4 represents the highest concentration). MDC‐CC, Malmö Diet and Cancer Cardiovascular Cohort; MPP, Malmö Preventive Project; PCT, procalcitonin.

In the pooled material, a high PCT was in a multivariate adjusted Cox‐regression model associated with more than a three‐fold increased risk of diagnosis‐verified liver disease during follow‐up, and the likelihood of developing diagnosis‐verified liver disease increased by 56% per 1 SD increment of log‐transformed PCT (Table 3). When subjects were separated into quartiles of PCT, the risk of liver disease showed a linear increase with increasing PCT quartiles (Table S1), and in a Kaplan–Meier plot with individuals split into quartiles of PCT concentration, a higher quartile was associated with an increased probability of liver disease (log‐rank test *p* < 0.001) (Figure 1).

**TABLE 3:** PCT as a predictor of liver disease in subjects from MPP and MDC‐CC without liver disease at baseline.

Diagnosis group *n* events/person‐years	Per 1 SD increase	*p*	High PCT^a^	*p*
Liver disease of any type and origin except viral hepatitis (*n* = 7717^b^) *n* = 119/139342	1.56 (1.32–1.85)	< 0.001	3.41 (2.07–5.63)	< 0.001
Fibrosis/cirrhosis of the liver of unknown origin (*n* = 7717^b^) *n* = 40/139930	1.81 (1.44–2.26)	< 0.001	9.66 (4.32–21.6)	< 0.001
Non‐specified liver failure (*n* = 7717^b^) *n* = 35/140019	1.70 (1.28–2.25)	< 0.001	5.39 (2.35–12.4)	< 0.001
Alcohol‐associated liver disease (*n* = 7717^b^) *n* = 28/139868	2.12 (1.60–2.79)	< 0.001	4.82 (1.91–12.2)	< 0.001

*Note:* Analyses adjusted for age, gender, BMI, hazardous alcohol consumption, HDL, LDL, triglycerides, lipid lowering treatment, prevalent diabetes, creatinine, prevalent hypertension, smoking.

Abbreviations: BMI, body mass index; HDL, high‐density lipoprotein cholesterol; LDL, low‐density lipoprotein cholesterol; MDC‐CC, Malmö Diet and Cancer Cardiovascular Cohort; MPP, Malmö Preventive Project; PCT, procalcitonin; SD, standard deviation.

^a^ High PCT defined as PCT > 0.05 ng/mL.

^b^ Total number of individuals in the analysis.

Different groups of diagnoses were also analysed separately and there were significant associations between elevated PCT and fibrosis/cirrhosis of the liver of unknown origin, non‐specified liver failure and alcohol‐associated liver disease during follow‐up (Table 3). Separate cohort‐specific sensitivity analyses, out of which the cohort‐specific analysis of MDC‐CC was additionally adjusted for CRP, showed similar effect estimates as the pooled analyses (Tables S2, S3).

Male sex, increasing BMI and smoking were all significantly associated with increased risk of liver disease in the multivariate adjusted model. There was, however, no significant interaction between PCT and male sex, BMI or smoking regarding the risk of liver disease, and therefore we did not perform subgroup analyses stratified by these variables.

## 4 Discussion

The key finding of this study is that elevated PCT is strongly and independently associated with future development of non‐viral liver disease both in pooled analyses of two population‐based cohorts, as well as in cohort‐specific analyses.

To our knowledge, this is the first study that investigates PCT concentration in the general population as a predictor of liver disease. This makes PCT interesting as a potential measure in future noninvasive scores that could be used to screen high‐risk individuals for liver disease. Apart from implications for the prediction of liver disease, our results point to a possible role of PCT in the pathophysiology behind the development of liver injury.

A small number of observations in a clinical setting have previously shown that patients with steatosis and steatohepatitis have similar PCT concentrations as healthy controls [25]. In cirrhotic patients, however, PCT concentrations seem to be higher compared to healthy controls [11]. In patients with acute hepatitis and no evidence of bacterial infection, PCT concentrations are shown to be elevated, with the highest concentrations seen in patients with acute liver failure, and a lower survival rate of patients with liver failure if PCT is ≥ 0.5 ng/mL [10]. PCT is also found to be elevated above the normal range in patients with advanced chronic liver disease [7]. Furthermore, elevated PCT has been associated with paracetamol‐induced liver injury and has been suggested as a predictive tool for toxic liver injury [26].

The pathophysiology behind why elevated PCT concentrations seem to be associated with liver disease is rather uninvestigated, but the gut‐liver‐axis concept, where the liver as an immune organ is described as a gatekeeper between the gut and the systemic circulation, may provide a basis for a hypothesis [27, 28]. Systemic inflammation, in which PCT may play a part, is believed to be a key factor in the pathophysiology of complications of advanced chronic liver disease. Previous studies have linked advanced chronic liver disease to increased bacterial translocation from the gut into the portal circulation [29, 30]. Bacterial endotoxins (pathogen‐associated molecular patterns, PAMPs) are released into the bloodstream due to reduced hepatic clearance. Hepatocyte damage, on the other hand, directly leads to the release of damage‐associated molecular pathways (DAMPs) into the bloodstream [31, 32, 33]. PAMPs and DAMPs, together with cytokines, are believed to trigger systemic inflammation in subjects with liver disease [34].

Experimental studies have shown that a proinflammatory environment may promote liver fibrosis, but the link between systemic inflammation and liver fibrinogenesis in humans is relatively uninvestigated [35, 36]. Among patients with advanced chronic liver disease, previous studies showed that PCT was independently associated with measures of liver fibrosis in both compensated and decompensated liver disease [12]. Most likely, the majority of individuals in our study did not have established cirrhosis at study baseline, and it is possible that fibrinogenesis accelerated due to elevated PCT as an early sign of systemic inflammation.

Moreover, PTC has been shown to have a direct negative effect on hepatocyte functions in vitro, such as proliferation, apoptosis, metabolism, integrity, microalbumin synthesis, and detoxification. This suggests that an increase in PCT concentrations as part of an inflammatory response due to liver injury could create a destructive cycle where the PCT itself maintains and enhances the hepatocyte damage. Hepatocytes also seem to be impaired even at very low concentrations of PCT compared to other cell types in vitro, suggesting that the liver is more susceptible to the direct toxic effects of PCT [14]. In relation to those findings, one may hypothesise that the elevated PCT at baseline in our study is not only an early sign of systemic inflammation linked to ongoing liver injury but also a direct cause of hepatocyte damage. If the liver is more susceptible to PCT toxicity than other organs, the concentrations at baseline in our study may be enough to induce hepatocyte damage.

MASLD is considered the liver component of metabolic disease, and elevated PCT has previously been associated with the metabolic syndrome [8]. One may thus speculate on the possibility that the association between elevated PCT and the development of liver disease is linked to metabolic disease. However, even after accounting for metabolic risk factors along with known risk factors for cardiovascular disease in our analyses, individuals with a high PCT had a three‐fold excess risk of developing liver disease compared with those without a high PCT.

The PCT concentration at study baseline was found to be higher in the MPP than in the MDC‐CC population. The MPP cohort consists of an older, predominantly male population with a lower mean eGFR than the MDC‐CC cohort, and we suggest that the higher PCT in MPP can be attributed to the fact that PCT concentration increases with age, is higher in men, and correlates to renal function decline [37]. The PCT concentration in MDC‐CC was, on the other hand, similar to PCT concentration found in a Dutch population‐based sample with a slightly younger mean age than MDC‐CC [8].

There are several strengths to our register‐based, prospective study. One important advantage is that the cohorts we have studied are relatively large and thus our results may be generalised to a middle‐aged population of northern Europe. We had access to detailed baseline data, which both allowed us to extract information about the general health status of the population and adjust for potential confounders which contributed to the reliability of the results. A long median follow‐up time of 14.8 years in MPP and 27.1 years in MDC‐CC made it possible to detect a sufficient number of cases of incident liver disease. The cases were identified by the Swedish National Patient Register, which is a reliable source that has been validated for liver‐related endpoints in previous studies [38].

However, there are also several limitations to our study. The Swedish National Patient Register did not cover hospital‐based outpatient care until 2001 and does not cover primary health care at all [24]. This means that most of our cases of liver disease were found among individuals admitted to inpatient care for various reasons, and we might have missed individuals diagnosed by their primary health care physician. This may also reflect the fact that we had a low number of diagnosis‐verified prevalent liver disease in the cohorts. Another limitation is that each separate diagnosis in this study was not validated through analyses of patient records. Also, despite access to detailed baseline data, we acknowledge that we cannot exclude residual confounding.

Due to the nature of chronic liver disease, many patients with liver fibrosis or early stages of compensated cirrhosis do not experience any symptoms or show any laboratory abnormalities. Therefore, they might not be diagnosed by their physician until they have advanced liver disease, which can take several years. This means there could be undiagnosed cases of incident liver disease during the follow‐up time. However, this would be expected to draw our conclusions towards the null. In line with this reasoning, the cases we detected most likely had advanced liver disease when they got their diagnosis and therefore could have had an early, asymptomatic stage of liver disease at baseline. However, since fibrosis can be reversible in an early stage of liver disease, it is important to investigate potential prognostic factors in these subjects as well.

Another limitation is that alcohol intake was assessed with a self‐administered questionnaire, which is a less reliable source than quantifiable, direct, or indirect biomarkers of alcohol consumption [39].

## 5 Conclusions

PCT is an independent predictive marker for incident non‐viral liver disease. In selected patients, it has the potential to be used as a risk marker leading to earlier detection of liver disease, and in turn, leading to earlier investigation and treatment of the underlying cause of liver injury. Our results also highlight the possibility of PCT as a direct cause of hepatocyte damage. If a causal relationship between elevated PCT concentration and hepatocyte damage could be proven, decreasing PCT concentration may be a future treatment target. Future studies to investigate the role of PCT in the pathophysiology of liver injury are warranted.

## Author Contributions

**Amanda Finnberg‐Kim:** statistical analysis and interpretation of data, drafting of the manuscript, review and editing of the manuscript. **Mats Pihlsgård:** statistical analysis and interpretation of data, review and editing of the manuscript. **Kristina Önnerhag:** study supervision, critical revision of the manuscript for important intellectual content, review and editing of the manuscript. **Olle Melander:** design and data collection of the cohorts included in the manuscript, critical revision of the manuscript for important intellectual content. **Sofia Enhörning:** study concept and design, obtained funding, study supervision, critical revision of the manuscript for important intellectual content, review and editing of the manuscript. All authors agree to be accountable for all aspects of this work and have given their final approval for this version to be published.

## Ethics Statement

The research was conducted in accordance with the Declaration of Helsinki, and study protocols were approved by the regional Ethics Committee of Lund University, Dnr. LU 51–90 and 85/2004.

## Consent

All participants provided written informed consent.

## Conflicts of Interest Statement

The authors decalre no conflicts of interest.

## Supporting information


Data S1.

## Data Availability

Data described in the manuscript are available upon reasonable request to each of the cohorts' principal investigators.
