# Supplementary material for: Procalcitonin as a Predictive Marker of Incident Liver Disease
Source: Liver Int. 2025 May 12;45(6):e70132. doi: 10.1111/liv.70132 (PMC12067363; doi:10.1111/liv.70132)
Supplement: Supplementary file 1 — Data S1. [file LIV-45-0-s001.docx]

**Supplementary material for:**

**Procalcitonin as a predictive marker of incident liver disease**

**Amanda Finnberg-Kim^1,2^, Mats Pihlsgård^3^, Kristina Önnerhag^2^, Olle Melander^1,4^, Sofia Enhörning^3,4^**

*^1^Department of Clinical Sciences in Malmö, Lund University, Malmö, Sweden; ^2^Department of Gastroenterology and Hepatology, Skåne University Hospital, Malmö, Sweden; ^3^Perinatal and Cardiovascular Epidemiology, Lund University Diabetes Centre, Department of Clinical Sciences in Malmö, Lund University, Malmö, Sweden; ^4^Department of Internal Medicine, Skåne University Hospital, Malmö, Sweden*

**Table of contents**

Supplementary tables S1-S3 page 2-4

**Supplementary tables S1-S3**

| **Table S1. PCT quartiles as a predictor of liver disease in subjects from MPP and MDC-CC without liver disease at baseline** | | | | |
| --- | --- | --- | --- | --- |
| **Diagnosis group**  **n events / person-years** | **Q2 vs Q1**  Hazard ratio (95% CI) | **Q3 vs Q1**  Hazard ratio (95% CI) | **Q4 vs Q1**  Hazard ratio (95% CI) | **P-value linear trend** |
| Liver disease of any type and origin except viral hepatitis (n=7717^†^)  n=119/139342 | 1.57 (0.93–2.67) | 1.63 (0.85–3.11) | 3.22 (1.67–6.20) | <0.001 |
| Fibrosis/cirrhosis of the liver of unknown origin (n=7717^†^)  n=40/139930 | 2.13 (0.78–5.83) | 1.59 (0.44–5.81) | 7.29 (2.21–24.0) | 0.001 |
| Non-specified liver failure (n=7717^†^)  n=35/140019 | 1.51 (0.48–4.75) | 2.38 (0.68–8.37) | 5.03 (1.40–18.1) | 0.008 |
| Alcohol-associated liver disease (n=7717^†^)  n=28/139868 | 8.23 (1.03–65.7) | 8.47 (0.95–75.5) | 21.7 (2.47–191.2) | 0.002 |
| ^†^ = Total number of individuals in the analysis  *Abbreviations:* PCT, procalcitonin; MPP, Malmö Preventive Project; MDC-CC, Malmö Diet and Cancer Cardiovascular cohort; Q, quartile; CI, confidence interval; BMI, body mass index; HDL, High-density lipoprotein cholesterol; LDL, Low-density lipoprotein cholesterol  PCT concentration by quartile, median (25 ^th^;75^th^), ng/mL: Q1: 0.013 (0.011-0.014); Q2: 0.019 (0.017-0.021); Q3: 0.031 (0.027-0.034); Q4: 0.050 (0.043-0.063)    Analyses adjusted for age, gender, BMI, hazardous alcohol consumption, HDL, LDL, triglycerides, lipid lowering treatment, prevalent diabetes, creatinine, prevalent hypertension, smoking. | | | | |

| **Table S2. PCT as a predictor of liver disease in subjects without liver disease at baseline in MDC-CC** | | | | |
| --- | --- | --- | --- | --- |
| **Diagnosis group**  **n events / person-years** | **Per 1 SD increase** | **p-value** | **High PCT**^‡^ | **p-value** |
| Liver disease of any type and origin except viral hepatitis (n=3888^†^)  n=70/91242 | 1.48 (1.19–1.85) | <0.001 | 4.18 (1.29–13.6) | 0.017 |
| Fibrosis/cirrhosis of the liver of unknown origin (n=3888^†^)  n=25/91682 | 1.66 (1.17–2.34) | 0.004 | 11.2 (2.49–49.9) | 0.002 |
| Non-specified liver failure (n=3888^†^)  n=15/91749 | 1.87 (1.30–2.69) | <0.001 | 15.7 (3.26–75.4) | <0.001 |
| Alcohol-associated liver disease (n=3888^†^)  n=15/91642 | 1.95 (1.29–2.97) | 0.002 | 12.1 (2.52–58.4) | 0.002 |
| ^†^ = Total number of individuals in the analysis  ^‡^ = High PCT defined as PCT >0.05 ng/mL  *Abbreviations:* PCT, procalcitonin; MDC-CC, Malmö Diet and Cancer Cardiovascular cohort; SD, Standard deviation; BMI, body mass index; HDL, High-density lipoprotein cholesterol; LDL, Low-density lipoprotein cholesterol; CRP, C-reactive protein  Analyses adjusted for age, gender, BMI, hazardous alcohol consumption, HDL, LDL, triglycerides, lipid lowering treatment, prevalent diabetes, creatinine, prevalent hypertension, smoking, CRP. | | | | |

| **Table S3. PCT as a predictor of liver disease in subjects without liver disease at baseline in MPP** | | | | |
| --- | --- | --- | --- | --- |
| **Diagnosis group**  **n events / person-years** | **Per 1 SD increase** | **p-value** | **High PCT**^‡^ | **p-value** |
| Liver disease of any type and origin except viral hepatitis (n=3829^†^)  n=49/48100 | 1.65 (1.27–2.15) | <0.001 | 3.51 (1.94–6.34) | <0.001 |
| Fibrosis/cirrhosis of the liver of unknown origin (n=3829^†^)  n=15/48248 | 2.38 (1.68–3.37) | <0.001 | 31.5 (6.86–144.5) | <0.001 |
| Non-specified liver failure (n=3829^†^)  n=20/48269 | 1.43 (0.89–2.30) | 0.136 | 3.76 (1.51–9.40) | 0.005 |
| Alcohol-associated liver disease (n=3829^†^)  n=13/48226 | 2.31 (1.55–3.45) | <0.001 | 3.87 (1.21–12.3) | 0.022 |
| ^†^ = Total number of individuals in the analysis  ^‡^ = High PCT defined as PCT >0.05 ng/mL  *Abbreviations:* PCT, procalcitonin; MDC-CC, Malmö Diet and Cancer Cardiovascular cohort; SD, Standard deviation; BMI, body mass index; HDL, High-density lipoprotein cholesterol; LDL, Low-density lipoprotein cholesterol  Analyses adjusted for age, gender, BMI, hazardous alcohol consumption, HDL, LDL, triglycerides, lipid lowering treatment, prevalent diabetes, creatinine, prevalent hypertension, smoking. | | | | |
